# Modulation of proliferation factors in lung adenocarcinoma with an analysis of the transcriptional consequences of genomic EGFR activation

**DOI:** 10.18632/oncotarget.27316

**Published:** 2019-12-10

**Authors:** Melanie Haas Kucherlapati

**Affiliations:** ^1^Department of Genetics, Harvard Medical School, Boston, Massachusetts 02115, USA; ^2^Department of Medicine, Division of Genetics, Brigham and Women’s Hospital, Boston, Massachusetts 02115, USA

**Keywords:** proliferation, mutation, transcription, EGFR, lung adenocarcinoma

## Abstract

Genes of the pre-replication, pre-initiation and replisome complexes duplicate the genome from many sites once in a normal cell cycle. This study examines complex components in lung adenocarcinoma (LUAD) closely, correlating changes in the genome and transcriptome with proliferation and overall survival. Molecular subtypes (The Cancer Genome Atlas (TCGA), 2014) based on copy number, DNA methylation, and mRNA expression had variable proliferation levels, the highest correlating with decreased survival. A pattern of increased expression typified by POLE2 and POLQ was found for multiple replication factors over thirty-seven tumor types. EGFR altered cases unanticipatedly inversely correlated with proliferation factor expression in LUAD, Colon adenocarcinoma, and Cancer Cell Line Encyclopedia cell lines, but not in glioblastoma or breast cancer. Activation mutations did not uniformly correlate with proliferation, most cases were pre-metastatic. A gene expression profile was identified, and pathway involvement considered. Significantly, results suggest EGFR over expression and activation are early alterations that likely stall the replication complex through PCNA phosphorylation creating replication stress responsible for DNA damage response and further mutation, but does not promote increased proliferation itself. An argument is presented that the mechanism driving lethality in this tumor cohort could differ from over proliferation seen in other LUAD.

## Introduction

It is well established that cancer is the result of accumulated genetic changes to tumor suppressor genes or oncogenes, and that these changes lead to uncontrolled cellular proliferation. This feature is important clinically, and many well established chemotherapeutic agents are designed to directly or indirectly inhibit DNA synthesis. Targeting the DNA is often effective, but has major disadvantages of toxicity due to non-specificity and predisposition to develop resistant clones. Cisplatin and carboplatin are two examples, often used in neoadjuvant and adjuvant chemotherapy regimens when treating patients with lung adenocarcinoma (LUAD). These reagents crosslink purine bases in DNA preventing replication and repair and promoting cell death. The choice to use them is made after careful initial assessment that takes into consideration risk factors, radiological appearance, tumor histology, and node involvement. Targeted therapies are now also available for several molecular classes of LUAD including EGFR mutation positive, ALK rearrangement positive, ROS1 rearrangement positive, BRAF V600E mutation positive, NTRK gene fusion positive, as well as anti-PD-L1 therapy [1].

Hypothetically, therapeutic reagents targeting DNA should be most effective for tumors that are highly proliferating and undergoing trans-lesion DNA synthesis. While uncontrolled replication can be initiated by changes involving cell cycle regulation, mitosis, and apoptosis among others, continued activity of genes in cellular proliferation is critical for the neoplastic state. This study focuses on genomic and transcriptional changes to proliferation genes across a LUAD cohort created by The Cancer Genome Atlas (TCGA) [2, 3], previously subtyped by them on the basis of copy number, DNA methylation, and mRNA expression. Selected genes coding for proteins involved in the pre-replication, pre-initiation, and replisome complexes were compared to evaluate the proliferation status of each of the subtypes. Since approximately 14% of the LUAD tumor cohort had Epidermal Growth Factor Receptor (EGFR) activation, and EGFR plays a known role in the replication of embryonic epidermal cells, it was included in the study as a LUAD proliferation factor.

The initial part of this study finds that subtype 2, 3, and 6 cases have highest expression of replication components and subtypes 1, 4, and 5 lowest; subtypes with highest expression have decreased survival. Evidence is presented that POLE and POLQ expression is elevated in subtype 2, and polymerase accessory subunits POLA2, POLD2, and POLE2 in subtypes 2 and 3. Comparing polymerase components across thirty-seven tumor types revealed a common subset with elevated transcriptional pattern typified by POLE2 and POLQ that included EXO1, MCM10, GINS2, CDT1, ORC6L, and BLM. Subtype 1 cases had increased expression of POLI, POLK, and POLL.

The second part of this study unexpectedly found that levels of EGFR expression overall were inversely proportional to the expression levels of multiple other important proliferation factors across the TCGA LUAD cohort. The same inverse relationship was found when examining EGFR expression in colon adenocarcinoma (COAD) and a cohort of cancer cell lines across several tissue types, but not in breast cancer (BRCA) or glioblastoma (GBB). To better understand the molecular process of EGFR activated LUAD, a search was made for genes altered in at least 50% of the activated cases. Twenty-one genes met the criterion, fifteen of which were found on the short arm of chromosome 7 proximal to EGFR. They included YKT6, MCPH1, UBE2D4, TP53, WIPI2, NUDCD3, PBXIP1, KLHL7, MRM2, HERPUD2, RNF216, FBXO42, FAM220A, URGCP, ZNF12, USP42, EXOC3, C7ORF26, VOPP1, ZDHHC4, and CLPTM1L.

Among the group CLPTM1L, PBXIP1, and URGCP like EGFR while showing increased expression over the EGFR cohort, inversely correlated with the expression of multiple key replication proteins over total LUAD. YKT6, KLHL7, FAM220A, and VOPP1 also had increased expression over the EGFR cohort but directly correlated with high expression of multiple proliferation genes. Altered cellular processes occurring with EGFR activation included increased PI3K/AKT/mTOR signaling and autophagy, cytokinesis and apoptosis impairment, exosome production, cytoskeletal changes, and multiple changes to proteins of the plasma membrane.

## Results

### Differential expression and overall survival

**Figure 1:**
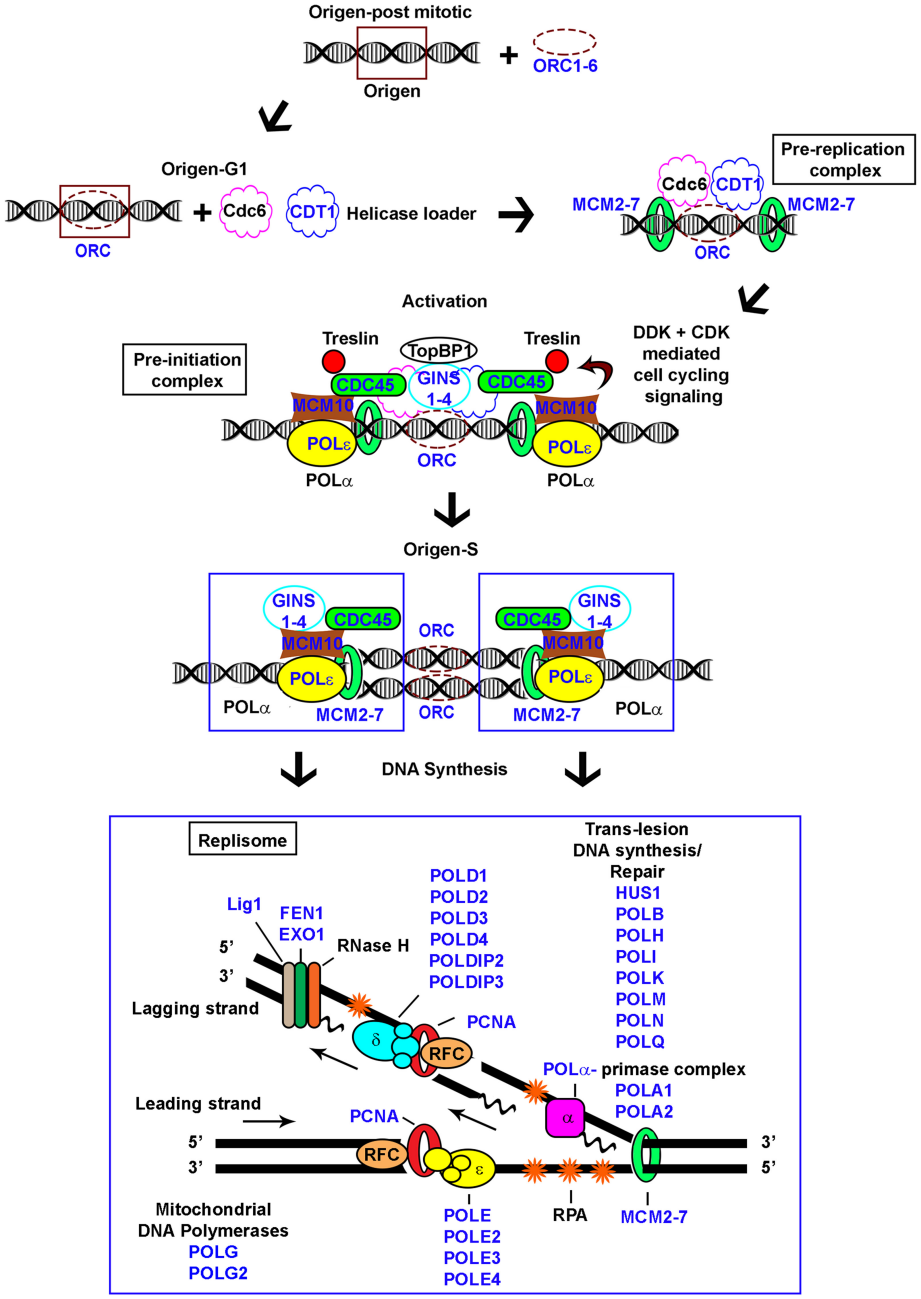
Schematic representation of eukaryotic replication. Pre-replication, pre-initiation, and replisome complexes depicted; genes examined in the study indicated (*blue*).

**Figure 2:**
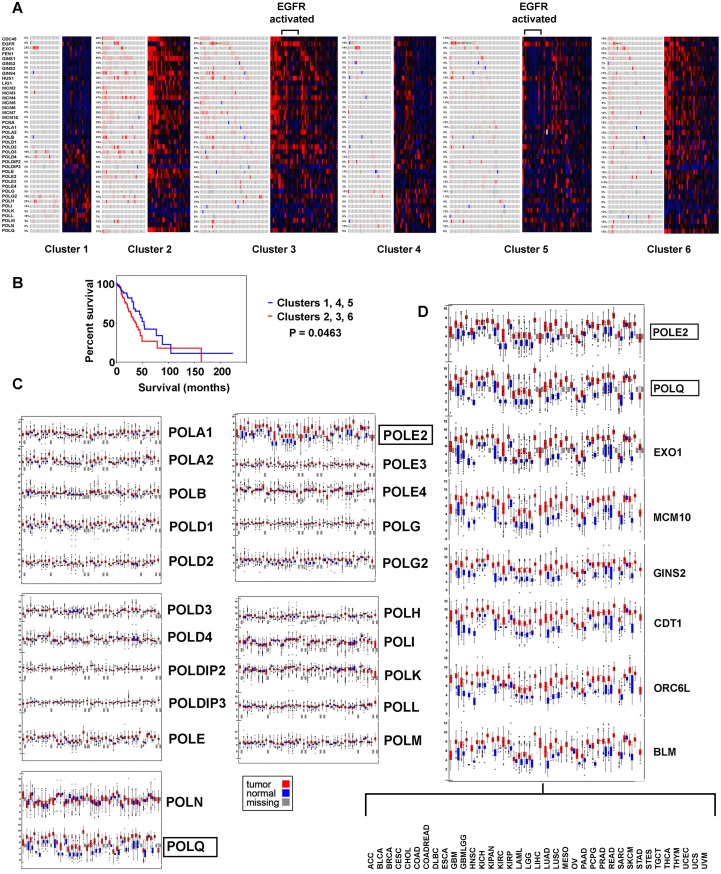
Replication genes examined for genomic and transcriptomic alteration in the context of LUAD subtypes, with survival analysis. (**A**) Cluster analysis is based on copy number, DNA methylation, and mRNA expression ([2, 3]), tumors with EGFR kinase activation are indicated. (**B**) Kaplan Meier survival plot, high versus low proliferating clusters. (**C**) Differential expression of polymerase components in thirty seven tumor types. (**D**) Replication genes found with common increased expression, thirty seven tumors.

The status of forty genes known to be involved in cellular proliferation was examined for genomic mutations and changes in expression over the LUAD tumor cohort (Figure 1) (Table 1). Alterations are presented in the context of six LUAD subtypes defined by cluster analysis based on copy number, DNA methylation, and mRNA expression (Figure 2A) [4]. Expression values in this image were calculated by cBioPortal [5, 6], which uses the value of the diploid fraction of the LUAD cohort as an estimated normal reference.

**Table 1:** Selected genes associated with DNA replication

Gene	Description	Location
CDC45	Cell Division Cycle 45	22q11.21
EGFR	Epidermal Growth Factor Receptor	7p11.2
EXO1	Exonuclease 1	1q43
FEN1	Flap Structure-Specific Endonuclease 1	11q12.2
GINS1	GINS Complex Subunit 1	20p11.21
GINS2	GINS Complex Subunit 2	16q24.1
GINS3	GINS Complex Subunit 3	16q21
GINS4	GINS Complex Subunit 4	8p11.21
HUS1	HUS1 Checkpoint Clamp Component	7p12.3
LIG1	DNA Ligase 1	19q13.33
MCM2	Minichromosome Maintenance Complex Component 2	3q21.3
MCM3	Minichromosome Maintenance Complex Component 3	6p12.2
MCM4	Minichromosome Maintenance Complex Component 4	8q11.21
MCM5	Minichromosome Maintenance Complex Component 5	22q12.3
MCM6	Minichromosome Maintenance Complex Component 6	2q21.3
MCM7	Minichromosome Maintenance Complex Component 7	7q22.1
MCM10	Minichromosome Maintenance Replication Initiation Factor	10p13
PCNA	Proliferating Cell Nuclear Antigen	20p12.3
POLA1	DNA Polymerase Alpha 1, Catalytic Subunit	Xp22.11-p21.3
POLA2	DNA Polymerase Alpha 2, Accessory Subunit	11q13.1
POLB	DNA Polymerase Beta	8p11.21
POLD1	DNA Polymerase Delta 1, Catalytic Subunit	19q13.33
POLD2	DNA Polymerase Delta 2, Accessory Subunit	7p13
POLD3	DNA Polymerase Delta 3, Accessory Subunit	11q13.4
POLD4	DNA Polymerase Delta 4, Accessory Subunit	11q13.2
POLDIP2	DNA Polymerase Delta Interacting Protein 2	17q11.2
POLDIP3	DNA Polymerase Delta Interacting Protein 3	22q13.2
POLE	DNA Polymerase Epsilon, Catalytic Subunit	12q24.33
POLE2	DNA Polymerase Epsilon 2, Accessory Subunit	14q21.3
POLE3	DNA Polymerase Epsilon 3, Accessory Subunit	9q32
POLE4	DNA Polymerase Epsilon 4, Accessory Subunit	2p12
POLG	DNA Polymerase Gamma, Catalytic Subunit	15q26.1
POLG2	DNA Polymerase Gamma 2, Accessory Subunit	17q23.3
POLH	DNA Polymerase Eta	6p21.1
POLI	Poly(ADP-Ribose) Polymerase 1	18q21.2
POLK	DNA Polymerase Kappa	5q13.3
POLL	DNA Polymerase Lambda	10q24.32
POLM	DNA Polymerase Mu	7p13
POLN	DNA Polymerase Nu	4p16.3
POLQ	DNA Polymerase Theta	3q13.33

LUAD subtypes with highest differential expression of replication factors (clusters 2, 3, and 6) versus lowest expression (clusters 1, 4, and 5) had decreased survival in Kaplan Meier survival plots (*P* = 0.0463) (Figure 2B). The most prominent feature between the groups is over expression of pre-replication and pre-initiation complex components and POLQ with relative under expression of POLI, POLK, POLL, and POLM in clusters 2, 3, and 6 implying these clusters have more “licensing”, origin firing and micro-homology end-joining. Subtypes 1, 4, and 5 had increased expression of POLH, POLI, POLK, and POLL, and POLM polymerases involved in trans-lesion DNA synthesis, double stranded break repair, and abasic site repair, suggesting the ability to carry out error prone DNA synthesis.

### Bimodal distribution and survival

mRNA levels for the forty genes were also examined for bimodal distribution above and below the LUAD tumor cohort average. Data are presented in the context of functional replication complexes (Tables 2–6). Mini-Chromosome (MCM) helicase proteins contribute to the pre-replication, pre-initiation, and replisome complexes. Components MCM 2, 3, 4, 6, and 7 were expressed above average consistently in subtype 2, differing significantly from subtype 1, 4, 5, and 6 (Table 2). MCM5 expression also was above average in subtype 2, but differed significantly only with subtype 1. The pre-initiation complex components CDC45, GINS1, GINS2, GINS3, GINS4, and MCM10 were found above the tumor average in subtype 2, differing significantly from subtype 1, 4, 5, and 6. Decreased survival was observed in Kaplan Meier survival curves for cases with MCM 2, 4, and 5, CDC45, GINS1, and MCM10 expression above the tumor cohort average (Table 3), in agreement with the general concept that high expression of proliferation genes correlates with decreased survival.

**Table 2:** Bimodal expression of pre-replication and pre-initiation complex components in LUAD

Gene	1 *n* = 22 Above/Below Average (%/%)	2 *n* = 32	3 *n* = 51	4 *n* = 32	5 *n* = 52	6 *n* = 41	2 vs 1	2 vs 3	2 vs 4	2 vs 5	2 vs 6
**Pre-Replication Complex** ****							**Fisher’s Exact (*P* value)**				
MCM2	4/18 (18/82)	25/7 (78/22)	31/20 (61/39)	13/19 (41/59)	20/32 (38/62)	19/22 (46/54)	0.000	0.148	0.005	0.001	0.008
MCM3	6/16 (27/73)	24/8 (75/25)	26/25 (51/49)	16/16 (50/50)	21/31 (40/60)	15/26 (37/63)	0.001	0.039	0.070	0.003	0.002
MCM4	4/18 (18/82)	27/5 (84/16)	35/16 (69/31)	13/19 (41/59)	17/35 (33/67)	20/21 (49/51)	0.000	0.127	0.001	0.000	0.003
MCM5	4/18 (18/82)	18/14 (56/44)	30/21 (59/41)	15/17 (47/53)	20/32 (38/62)	24/17 (59/41)	0.010	0.824	0.617	0.122	1.000
MCM6	4/18 (18/82)	24/8 (75/25)	37/14 (73/27)	11/21 (34/66)	22/30 (42/58)	23/18 (56/44)	0.000	1.000	0.002	0.006	0.139
MCM7	5/17 (23/77)	25/7 (78/22)	32/19 (63/37)	12/20 (38/62)	16/36 (31/69)	16/25 (39/61)	0.000	0.155	0.002	0.000	0.001
**Pre-Initiation Complex**											
CDC45	4/18 (18/82)	27/5 (84/16)	35/16 (69/31)	8/24 (25/75)	14/38 (27/73)	28/13 (68/32)	0.000	0.127	0.000	0.000	0.171
GINS1	2/20 (9/91)	28/4 (88/12)	32/19 (63/37)	8/24 (25/75)	22/30 (42/58)	27/14 (66/34)	0.000	0.022	0.000	0.000	0.054
GINS2	5/17 (23/77)	24/8 (75/25)	35/16 (69/31)	10/22 (31/69)	23/29 (44/56)	20/21 (49/51)	0.000	0.623	0.001	0.007	0.031
GINS3	3/19 (14/86)	21/11 (66/34)	28/23 (55/45)	10/22 (31/69)	22/30 (42/58)	22/19 (54/46)	0.000	0.368	0.012	0.045	0.345
GINS4	2/20 (9/91)	28/4 (88/12)	29/22 (57/43)	6/26 (19/81)	15/37 (29/71)	18/23 (44/56)	0.000	0.672	0.000	0.000	0.000
MCM10	3/19 (14/86)	28/4 (88/12)	33/18 (65/35)	8/24 (25/75)	15/37 (29/71)	25/16 (61/39)	0.000	0.024	0.000	0.000	0.017

**Table 3:** Bimodal survival for pre-replication and pre-initiation complex components

Gene	Total Above Average	Total Below Average	Kaplan Meier Worse Survival	Log-rank
**Pre-Replication Complex**				
MCM2	94	109	Above	0.031
MCM3	92	111	NA	0.215
MCM4	99	104	Above	0.015
MCM5	94	109	Above	0.004
MCM6	103	100	NA	0.070
MCM7	89	114	NA	0.129
**Pre-Initiation Complex**				
CDC45	96	107	Above	0.011
GINS1	100	103	Above	0.013
GINS2	99	104	NA	0.207
GINS3	90	113	NA	0.132
GINS4	84	119	NA	0.146
MCM10	93	110	Above	0.039

**Table 4:** Bimodal expression of replisome factors

**Gene**	**1 *n* = 22 Above/Below Average (%/%)**	**2 *n* = 32**	**3 *n* = 51**	**4 *n* = 32**	**5 *n* = 52**	**6 *n* = 41** ****	**2 vs 1**	**2 vs 3**	**2 vs 4**	**2 vs 5**	**2 vs 6**
**Pre-Replication Complex**							**Fisher’s Exact (*P* value)**				
PCNA	6/16 (27/73)	24/8 (75/25)	24/27 (47/53)	10/22 (31/69)	22/30 (42/58)	21/20 (51/49)	0.001	0.014	0.001	0.006	0.053
FEN1	5/17 (23/77)	27/5 (84/16)	31/20 (61/39)	11/21 (34/66)	17/35 (33/67)	21/20 (31/49)	0.000	0.028	0.000	0.000	0.006
EXO1	5/17 (23/77)	26/6 (81/19)	34/17 (67/33)	11/21 (34/66)	17/35 (33/67)	26/15 (63/37)	0.000	0.209	0.029	0.000	0.121
LIG1	7/15 (32/68)	22/10 (69/31)	31/20 (61/39)	16/16 (50/50)	25/27 (48/52)	20/21 (49/51)	0.012	0.491	0.202	0.074	0.10
POLA1	7/15 (32/68)	20/12 (63/38)	27/24 (53/47)	20/12 (63/38)	29/23 (56/44)	19/22 (46/54)	0.051	0.500	1.000	0.650	0.238
POLA2	3/19 (14/86)	23/9 (72/28)	32/19 (63/37)	11/21 (34/66)	15/37 (29/71)	21/20 (51/49)	0.000	0.478	0.005	0.000	0.094
POLB	2/20 (9/91)	17/15 (53/47)	28/23 (55/45)	19/13 (59/41)	19/33 (37/63)	13/28 (32/68)	0.001	1.000	0.801	0.175	0.093
POLD1	6/16 (27/73)	20/12 (63/37)	35/16 (69/31)	17/15 (53/47)	23/29 (44/56)	23/18 (56/44)	0.014	0.636	0.613	0.120	0.64
POLD2	9/13 (41/59)	19/13 (59/41)	40/11 (78/22)	10/22 (31/69)	16/36 (31/69)	19/22 46/54)	0.268	0.083	0.044	0.013	0.347
POLD3	10/12 (45/55)	24/8 (75/25)	21/30 (41/59)	17/15 (53/47)	27/25 (52/48)	19/22 (46/54)	0.044	0.003	0.117	0.041	0.017
POLD4	15/7 (68/32)	10/22 (31/69)	29/22 (57/43)	14/18 (44/56)	18/34 (35/65)	23/18 (56/44)	0.012	0.026	0.439	0.815	0.058
POLDIP2	15/7 (68/32)	19/13 (59/41)	29/22 (57/43)	15/17 (47/53)	19/33 (37/63)	15/26 (37/63)	0.576	1.000	0.453	0.046	0.062
POLDIP3	14/8 (64/36)	17/15 (53/47)	27/24 (53/47)	25/7 (78/22)	33/19 (63/37)	12/29 (29/71)	0.577	1.000	0.064	0.257	0.054
POLE	10/12 (45/55)	28/4 (88/13)	28/23 (55/45)	12/20 (38/63)	25/27 (48/52)	20/21 (49/51)	0.002	0.002	0.000	0.000	0.001
POLE2	4/18 (18/82)	28/4 (88/13)	44/7 (86/14)	9/23 (28/72)	14/38 (27/73)	26/15 (63/37)	0.000	1.000	0.000	0.000	0.031
POLE3	7/15 (32/68)	17/15 (53/47)	29/22 (57/43)	15/17 (47/53)	25/27 (48/52)	20/21 (49/51)	1.000	0.822	0.803	0.823	0.815
POLE4	8/14 (36/64)	19/13 (59/41)	24/27 (47/53)	14/18 (44/56)	18/34 (35/65)	13/28 (32/68)	0.166	0.367	0.317	0.041	0.032
POLG	16/6 (73/27)	21/11 (66/34)	25/26 (49/51)	24/8 (75/25)	27/25 (52/48)	22/19 (54/46)	0.767	0.176	0.585	0.261	0.345
POLG2	10/12 (45/55)	17/15 (53/47)	35/16 (69/31)	16/16 (50/50)	21/31 (40/60)	12/29 (29/71)	0.782	0.170	1.000	0.270	0.054
POLH	12/10 (55/45)	16/16 (50/50)	27/24 (53/47)	20/12 (63/38)	31/21 (60/40)	15/26 (37/63)	0.787	0.825	0.450	0.498	0.218
POLI	19/3 (96/14)	19/13 (59/41)	22/29 (43/57)	18/14 (56/44)	22/30 (42/58)	15/26 (37/63)	0.039	0.180	1.000	0.178	0.062
POLK	14/8 (64/36)	10/22 (31/69)	24/27 (47/53)	19/13 (59/41)	34/18 (65/35)	21/20 (51/49)	0.027	0.176	0.044	0.003	0.101
POLL	19/13 (86/14)	18/14 (56/44)	20/31 (39/61)	21/11 (66/34)	28/24 (54/46)	15/26 (37/63)	0.035	0.175	0.609	1.000	0.105
POLM	16/6 (73/27)	16/16 (50/50)	24/27 (47/53)	16/16 (50/50)	25/27 (48/52)	19/22 (46/54)	0.158	0.825	1.000	1.000	0.816
POLN	5/17 (23/77)	15/17 (47/53)	17/34 (33/67)	9/23 (28/72)	23/29 (44/56)	18/23 (44/56)	0.091	0.252	0.196	0.825	0.817
POLQ	4/18 (18/82)	23/9 (72/28)	23/28 (45/55)	6/26 (19/81)	14/38 (27/73)	22/19 (54/46)	0.000	0.023	0.000	0.000	0.148

**Table 5:** Polymerase accessory subunits have high expression in subtypes 2 and 3

Gene	1 *n* = 22 Above/Below Average (%/%)	2 *n* = 32	3 *n* = 51	4 *n* = 32	5 *n* = 52	6 *n* = 41	3 vs 1	3 vs 2	3 vs 4	3 vs 5	3 vs 6
**Pre-Replication Complex**							**Fisher’s Exact (*P* value)**				
POLA2	3/19 (14/86)	23/9 (72/28)	32/19 (63/37)	11/21 (34/66)	15/37 (29/71)	21/20 (51/49)	0.000	0.478	0.014	0.000	0.000
POLD2	9/13 (41/59)	19/13 (59/41)	40/11 (78/22)	10/22 (31/69)	16/36 (31/69)	19/22 (46/54)	0.003	0.083	0.000	0.000	0.002
POLE2	4/18 (18/82)	28/4 (88/13)	44/7 (86/14)	9/23 (28/72)	14/38 (27/73)	26/15 (63/37)	0.000	1.000	0.000	0.000	0.014

**Table 6:** Bimodal survival for replisome factors

Gene	Total Cases Above Average	Total Cases Below Average	Kaplan Meier Worse Survival	Log-rank (Mantel-Cox)	Gehan-Breslow-Wilcoxon Worse Survival	Gehan-Breslow-Wilcoxon test
**Replisome**						
PCNA	103	92	NA	0.156	Above	0.028
FEN1	90	105	NA	0.084	Above	0.020
EXO1	96	99	Above	0.002	Above	0.003
LIG1	99	96	NA	0.156	Above	0.028
POLA1	100	95	NA	0.634	NA	0.273
POLA2	82	113	NA	0.106	Above	0.038
POLB	81	114	NA	0.132	NA	0.391
POLD1	103	92	NA	0.291	NA	0.067
POLD2	93	102	Above	0.005	Above	0.002
POLD3	99	96	NA	0.173	Above	0.020
POLD4	89	106	NA	0.661	NA	0.721
POLDIP2	90	105	NA	0.059	NA	0.242
POLDIP3	103	92	NA	0.729	NA	0.638
POLE	97	98	NA	0.771	NA	0.270
POLE2	92	103	NA	0.089	Above	0.036
POLE3	88	107	NA	0.608	NA	0.250
POLE4	76	119	NA	0.417	NA	0.506
POLG	110	85	NA	0.306	NA	0.512
POLG2	92	103	NA	0.994	NA	0.996
POLH	103	92	NA	0.051	NA	0.285
POLI	99	96	NA	0.312	NA	0.991
POLK	103	92	NA	0.591	NA	0.386
POLL	101	94	NA	0.083	NA	0.428
POLM	97	98	NA	0.927	NA	0.477
POLN	73	122	NA	0.237	Above	0.046
POLQ	72	123	NA	0.097	Above	0.020

Replisome complexes duplicate DNA on leading and lagging strands (Figure 1). PCNA is a major component of this complex forming a sliding ring structure that attracts and tethers many other replicative proteins, Flap endonuclease (FEN1) is an integral component of lagging strand synthesis. PCNA and FEN1 expression were significantly above tumor average in subtype 2, as were EXO1 and LIG1 (Table 4). Examination of components comprising polymerases POLA, POLB, POLD, POLE, POLG, POLH, POLI, POLK, POLL, POLM, POLN, and POLQ showed POLE (catalytic subunit) expression elevated significantly in subtype 2 (Table 4); accessory subunits (POLA2, POLD2, and POLE2) were significantly elevated in subtypes 2 and 3, potentially signifying a function other than structural for their respective polymerase complexes in cancer (Table 5). POLQ expression was significantly elevated in subtype 2. Subtype 1 cases had increased expression of POLI, POLK, and POLL relative to subtype 2 (Table 4). Cases with POLD2 and EXO1 expression above the tumor average had decreased survival by Log-rank (Table 6). Applying Gehan-Breslow-Wilcoxon test indicated cases with FEN1, POLD3, POLQ, LIG1, PCNA, POLE2, POLA2, and POLN expression above the tumor average tended towards decreased survival.

### Proliferation genes highly transcribed in cancer

A common pattern of increased expression for POLE2 and POLQ in LUAD were found across thirty-seven tumor types [7]. Searching for other proliferation genes resulted in finding EXO1, MCM10, GINS2, CDT1, ORC6L, and BLM had the same pattern indicating they are most likely necessarily highly transcribed in cancer (Figure 2C and 2D).

### EGFR expression and proliferation

**Figure 3:**
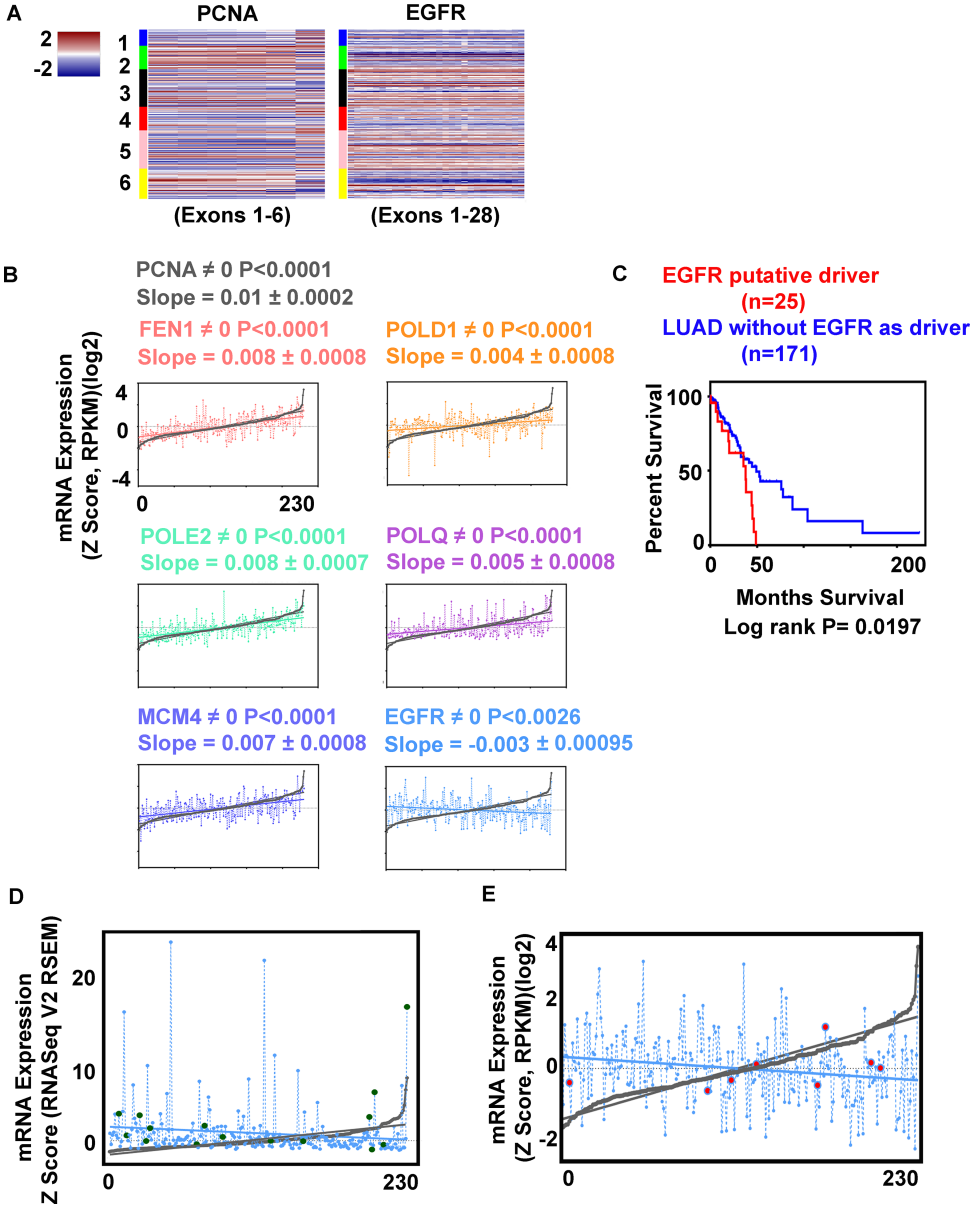
EGFR expression correlates inversely with replication genes expression. (**A**) Heatmaps comparing PCNA and EGFR mRNA expression for each exon, cases arranged in LUAD clusters (1–6). (**B**) Regression analysis. Cases arranged by PCNA mRNA minimum to maximum values, and compared to second gene expression; significance by *P* value. FEN1 (*pink*), POLD1 (*orange*), POLE2 (*green*), POLQ (*magenta*), MCM4 (*purple*), EGFR (*blue*). (**C**) Kaplan Meier survival plot, significance by Log rank test; LUAD with EGFR activated kinase (*red*) no activation (*blue*) (for case identification, see Supplementary Table 1). (**D**) Differential expression of EGFR (*blue*) missense mutations (*green*) (putative drivers, *n* = 15) compared to PCNA (*min-max*) curve (*grey*). (**E**) Relative expression of EGFR (*blue*) cases with distant metastasis (*red*) compared to PCNA curve (*grey*).

EGFR activated cases were found in multiple LUAD subtypes, with low differential expression of pre-replication, pre-initiation, and replisome complex factors (Figure 2A). Comparing EGFR and PCNA mRNA heatmaps (exonic level) side by side, suggested an inverse relationship from one subtype to the next (Figure 3A), an impression supported by cBioPortal differential expression data. These findings motivated arranging cases by PCNA mRNA expression (minimum to maximum), and interrogating other gene(s) expression relative to the curve. FEN1, POLD1, POLE2, POLQ, and MCM4, proteins that physically interact with PCNA or function close by, had expression curves in direct correlation to the DNA clamp. EGFR mRNA expression was inversely proportional to all (Figure 3B). PDGFRA (4q12), another cell surface tyrosine kinase receptor, similar to EGFR also had expression inversely correlated to the PCNA curve (data not shown). A Kaplan Meier survival plot comparing all cases with putative EGFR driver mutations to cases without EGFR alteration showed significant decreased survival for patients with putative driver mutations (Figure 3C) (Supplementary Table 1). Cases with EGFR missense mutations that were putative drivers did not cluster at any one point when arranged lowest to highest proliferation markers (Figure 3D) suggesting EGFR activation does not directly lead to increased proliferation in LUAD. This is supported by the fact that the twenty-eight EGFR tyrosine kinase activated cases also localize to the multiple LUAD subtypes predominantly 3, 5, and 6 (Figure 2A). Eight LUAD cases with distant metastasis tended towards higher placement on the PCNA proliferation curve, but not highest (Figure 3E); only one of these cases was also EGFR activated. Altogether, these results seem to support idea that the lethality in LUAD achieved through the EGFR activation pathway, and the lethality in LUAD achieved by increased levels proliferation are different mechanistically.

**Figure 4:**
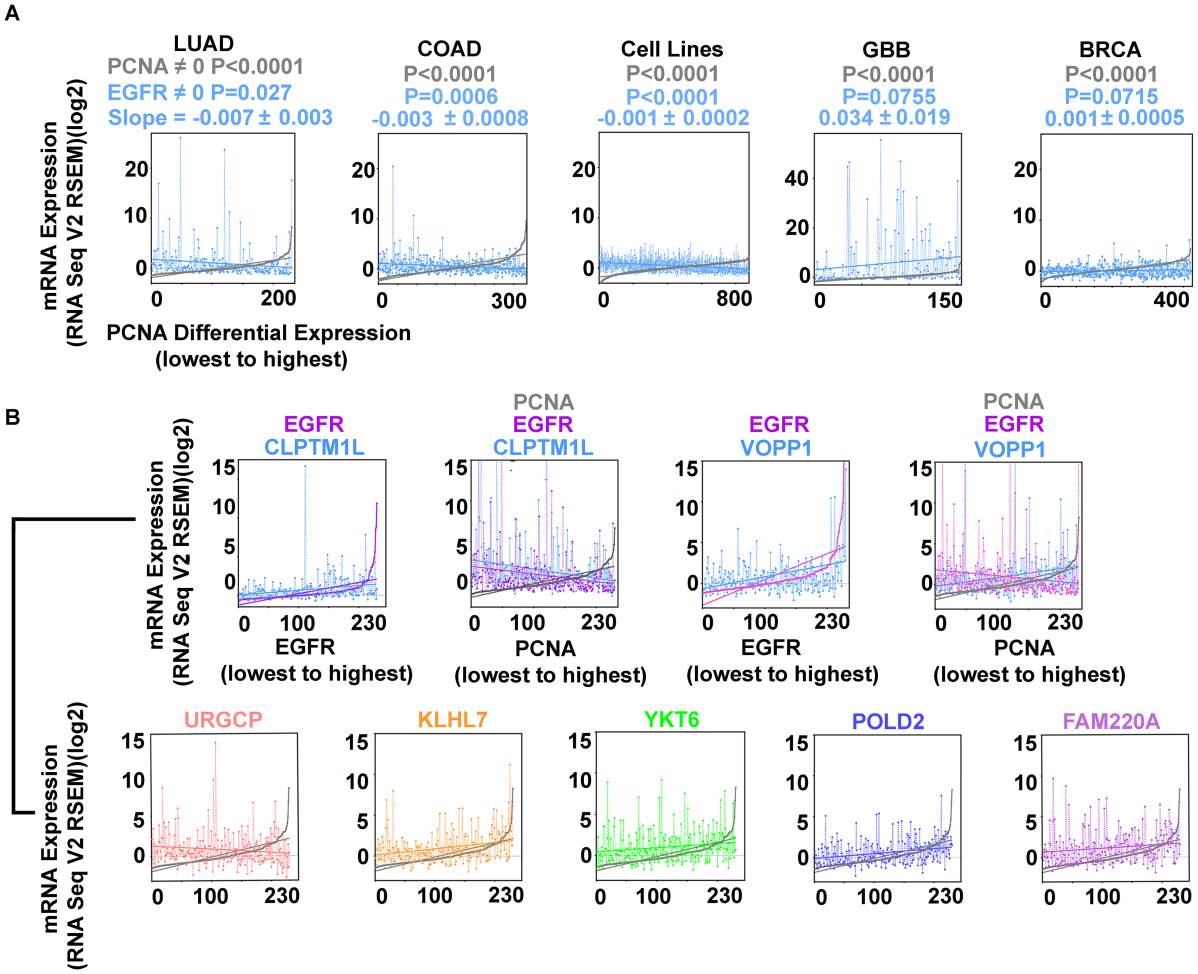
Inverse correlation of EGFR and PCNA expression are found in LUAD, COAD, and cell lines from multiple tumor types (Cancer Cell Line Encyclopedia; (CCLE); *n* = 877; Supplementary Table 2) but not in glioblastoma (GBB) or breast cancer (BRCA). (**A**) EGFR (*blue*) and PCNA (*min-max*) curve (*grey*); LUAD, COAD, CCLE cell lines, GBB, and BRCA. (**B**) EGFR overexpression accompanies secondary gene overexpression that can vary proportionally with proliferation. EGFR (*magenta*), CLPTM1L (*blue*) cases arranged by EGFR expression (*min-max*) illustrating direct proportion; EGFR (*magenta*) CLPTM1L (*blue*) and PCNA (*grey*) cases rearranged by PCNA expression (*min-max*) illustrating indirect proportion to proliferation. EGFR (*magenta*), VOPP1 (*blue*) arranged by EGFR expression (*min-max*); EGFR (*magenta*), VOPP1 (*blue*), and PCNA (*grey*) cases rearranged by PCNA expression (*min-max*) illustrating simultaneous VOPP1 direct proportion and EGFR indirect proportion to proliferation. URGCP (*pink*), KLHL7 (*orange*), YKT6 (*green*), POLD2 (*purple*), FAM220A (*magenta*), all arranged by PCNA expression (*min-max*) (*grey*).

Examining the behavior of EGFR differential expression to proliferation markers in COAD, BRCA, GBB, and the Cancer Cell Line Encyclopedia (from the Broad Institute and Novartis, 877 samples) [8, 9] (Supplementary Table 2) permitted identification of the inverse relationship in COAD and in the cell lines, but not in GBB or BRCA (Figure 4A). GBB had higher EGFR expression levels overall compared to LUAD, COAD, BRCA, and the cell line cohort.

### Genes involved in EGFR activated cases

**Figure 5:**
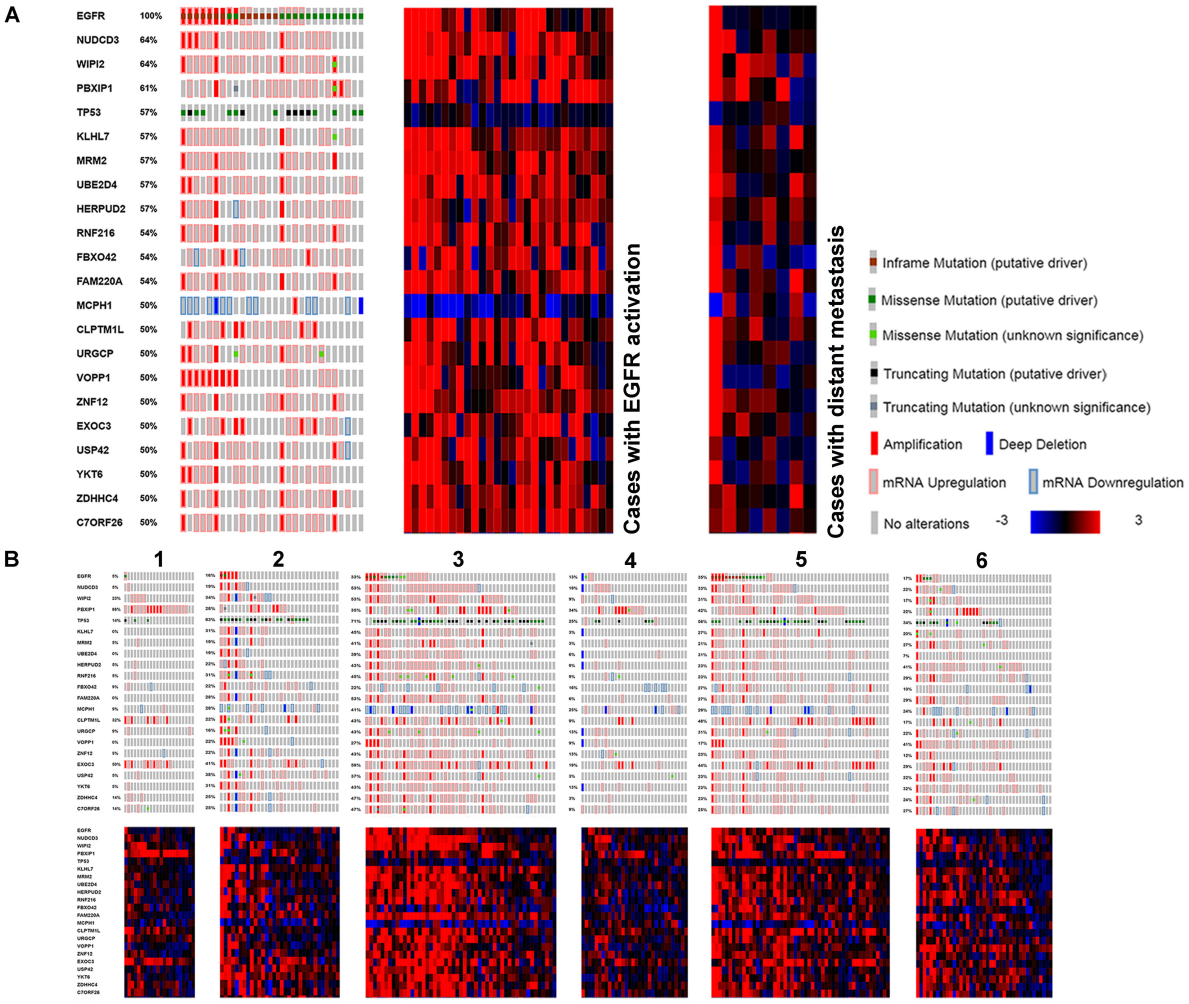
Genes found with genomic and transcriptomic alterations in EGFR activated LUAD, with high frequency. (**A**) Compared to cases with distant metastasis. (**B**) Compared to TCGA subtypes.

Twenty-one genes with genomic and expression alterations in at least 50% of EGFR tyrosine kinase activated cases were identified (Table 7). They were examined over LUAD subtypes 1-6, and in cases with distant metastases (Figure 5). Clusters 3 and 5 containing most of the EGFR tyrosine kinase activated cases were most similar, metastatic cases were less so. TP53 and MCPH1 both had low differential expression. TP53 had additional frequent genomic mutation (at DNA binding domain, 46.5%) MCPH1 did not (0.9%). MCPH1 expression was altered across the entire LUAD cohort (28%) including cases with increased expression. Mutual exclusivity testing for MCPH1 (low expression) and EGFR revealed they co-occurred significantly (*P* = 0.004). YKT6, UBE2D4, WIPI2, NUDCD3, PBXIP1, KLHL7, MRM2, HERPUD2, RNF216, FBXO42, FAM220A, URGCP, ZNF12, USP42, EXOC3, C7ORF26, VOPP1, ZDHHC4, and CLPTM1L were more highly expressed. Fifteen of these had cytological locations on the short arm of chromosome 7 (Table 7). The dysregulation of so many other genes proximal to EGFR’s cytological location suggests an epigenetic event affecting transcription as an early alteration in these tumors.

**Table 7:** Expression alterations in EGFR activated cases and their relationship to proliferation

Gene	EGFR Cases (%)	Cytogenetic Band	Slope	+/–	*P* value (Slope ≠ 0)	Slope relative to PCNA
EGFR	100	7p11.2	–0.0074	0.003	0.027	Negative
YKT6	50	7p13	0.0050	0.002	0.010	Positive
MCPH1	50	8p23.1	0.0029	0.002	0.131	None
UBE2D4	57	7p13	0.0014	0.002	0.425	None
TP53	57	17p13.1	–0.0003	0.001	0.668	None
WIPI2	64	7p22.1	–0.0011	0.002	0.593	None
NUDCD3	64	7p13	–0.0012	0.002	0.607	None
PBXIP1	61	1q21.3	–0.0129	0.002	0.000	Negative
KLHL7	57	7p15.3	0.0077	0.002	0.000	Positive
MRM2	57	7p22.3	0.0033	0.002	0.065	None
HERPUD2	57	7p14.2	0.0019	0.002	0.326	None
RNF216	54	7p22.1	–0.0039	0.002	0.087	None
FBXO42	54	1p36.13	–0.0013	0.002	0.432	None
FAM220A	54	7p22.1	0.0045	0.002	0.023	Positive
URGCP	50	7p13	–0.0043	0.002	0.029	Negative
ZNF12	50	7p22.1	–0.0029	0.002	0.087	None
USP42	50	7p22.1	–0.0014	0.002	0.451	None
EXOC3	50	5p15.33	–0.0040	0.004	0.353	None
C7ORF26	50	7p22.1	0.0003	0.002	0.883	None
VOPP1	50	7p11.2	0.0108	0.004	0.009	Positive
ZDHHC4	50	7p22.1	0.0034	0.002	0.092	None
CLPTM1L	50	5p15.33	–0.0114	0.003	0.000	Negative
GLI3	39	7p14.1	–0.0018	0.003	0.501	None
GNA12	36	7p22.3-p22.2	–0.0002	0.002	0.905	None
HUS1	29	7p12.3	0.0012	0.001	0.381	None
POLD2	29	7p13	0.0061	0.001	0.000	Positive
POLM	21	7p13	–0.0007	0.001	0.610	None
TWIST1	14	7p21.1	0.0021	0.001	0.052	None

### Genes involved in EGFR activated cases in relation to proliferation

Comparing EGFR relevant gene expression to the PCNA expression curve, URGCP, PBXIP1, and CLPTM1L have inverse relationships to proliferation, while VOPP1, YKT6, KLHL7, and FAM220A are direct (Table 7, Figure 4B). VOPP1 (also known as Vesicular, Overexpressed in cancer, Prosurvival Protein 1 or EGFR-Co-amplified and Overexpressed Protein) expression is shown first in relation to EGFR expression, then to PCNA expression. VOPP1 is overexpressed in the most highly proliferative cases, with lowest EGFR expression, the finding was confirmed in cBioPortal. CLPTM1L, a gene coding for a membrane protein that when over expressed in cisplatin-sensitive cells causes apoptosis, is over expressed in EGFR activated cases that are the least proliferative.

### Survival correlations for EGFR relevant genes

**Figure 6:**
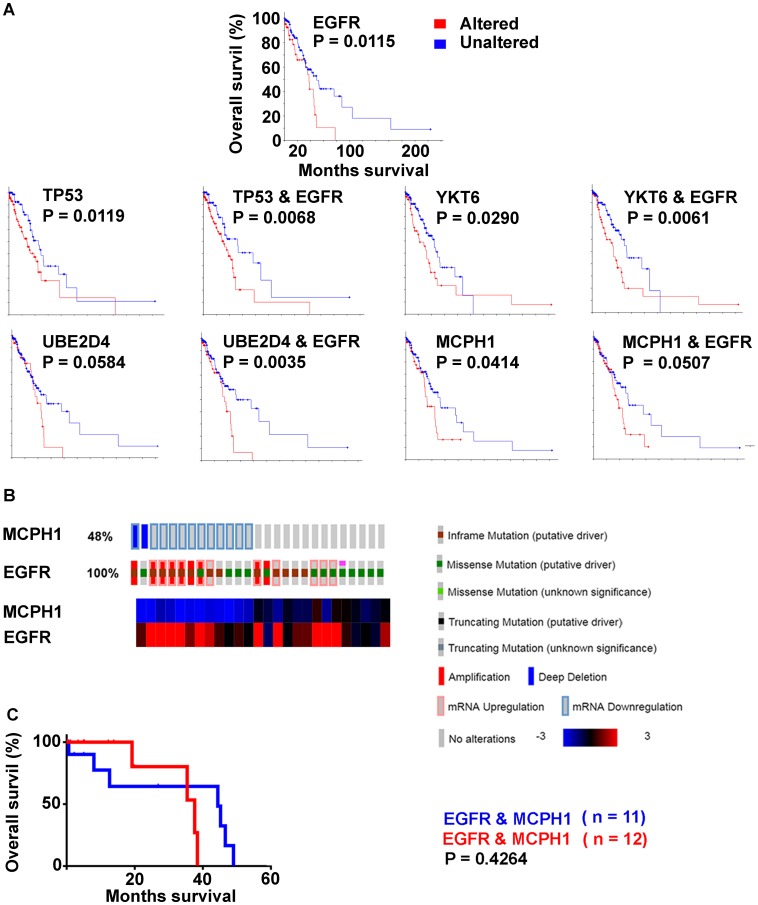
Decreased overall survival (Kaplan Meier survival plots, Log rank; LUAD with survival data, *n* = 197) by genes with high frequency alterations in EGFR activated cases. (**A**) EGFR altered (*red*) unaltered (*blue*). TP53, YKT6, UBE2D4, MCPH1 with and without EGFR alteration. (**B**) MCPH1 and EGFR, EGFR activated cases only. (**C**) Kaplan Meier survival plot; reduced versus normal expression.

Genes implicated in the EGFR subtype were examined for overall survival across the LUAD tumor cohort (*n* = 230) using the Kaplan-Meier tool in cBioPortal (Figure 6). Cases with alteration in the genome and/or expression levels to TP53, YKT6, UBE2D4, and MCPH1 had decreased survival when compared to cases that did not. Cases with EGFR alteration in addition to each of these genes showed increased significance (Log rank) for YKT6, UBE2D4, and TP53. MCPH1 had decreased significance (Log rank) when in combination with EGFR.

A matrix table was set up to identify combinatorial alterations to TP53, YKT6, UBE2D4, and MCPH1 for individual EGFR activated cases (Table 8). Kaplan-Meier survival curves were calculated for each combination over the entire LUAD cohort, and sorted on the basis of Log-rank outcome. MCPH1 under expression correlated with loss of significance between curves for all gene permutations, with decrease in median survival seen in the “unaltered” curve. In EGFR activated cases alone, those with MCPH1 reduced expression (*n* = 11) were compared with MCPH1 normal expression (*n* = 12) when survival data was available, the curves were not significantly different from each other (*P* = 0.4264) (Figure 6).

**Table 8:** Combinatory alterations to TP53, YKT6, UBE2D4, and MCPH1 in EGFR activated cases and their survival

**TCGA ID (*n* = 26)**	**EGFR**^**a**^	**TP53**^**a**^	**YKT6**^**a**^	**UBE2D4**^**a**^	**MCPH1**^**a**^	**(Logrank)**^**b**^ **(*n* = 230)**	**Median survival altered (mo)**	**Altered Total/Died**	**Median survival unaltered (mo)**	**Unaltered Total/Died**
TCGA-44-2661-01	X	O	O	X	O	0.003	35.5	61/26	53.3	142/37
TCGA-67-6217-01	X	O	X	X	O	0.004	32.7	86/37	76.2	117/26
TCGA-49-4494-01	X	O	X	X	O	0.004	32.7	86/37	76.2	117/26
TCGA-78-7147-01	X	X	O	X	O	0.005	37.7	119/44	76.2	84/19
TCGA-05-5423-01	X	X	O	X	O	0.005	37.7	119/44	76.2	84/19
TCGA-38-4627-01	X	O	X	O	O	0.006	32.7	79/34	52.5	124/29
TCGA-55-7573-01	X	X	O	O	O	0.007	38.5	113/42	76.2	90/21
TCGA-05-4382-01	X	X	O	O	O	0.007	38.5	113/42	76.2	90/21
TCGA-75-6207-01	X	X	X	X	O	0.010	37.7	129/47	76.2	74/16
TCGA-78-7155-01	X	X	X	X	O	0.010	37.7	129/47	76.2	74/16
TCGA-50-5944-01	X	O	O	O	O	0.012	37.7	45/19	49.2	158/44
TCGA-75-7025-01	X	O	O	O	O	0.012	37.7	45/19	49.2	158/44
TCGA-91-6835-01	X	O	O	O	O	0.012	37.7	45/19	49.2	158/44
TCGA-38-6178-01	X	X	X	O	O	0.015	37.7	126/46	76.2	77/17
TCGA-75-6212-01	X	O	O	X	X	0.056	35.5	93/33	53.3	110/30
TCGA-49-4501-01	X	O	O	X	X	0.056	35.5	93/33	53.3	110/30
TCGA-67-3772-01	X	O	O	X	X	0.056	35.5	93/33	53.3	110/30
TCGA-91-6847-01	X	X	O	O	X	0.060	38.5	130/45	53.3	73/18
TCGA-64-1681-01	X	X	O	O	X	0.060	38.5	130/45	53.3	73/18
TCGA-49-4490-01	X	X	X	X	X	0.137	38.5	143/48	53.3	60/15
TCGA-69-7760-01	X	X	X	X	X	0.137	38.5	143/48	53.3	60/15
TCGA-05-4402-01	X	X	X	X	X	0.137	38.5	143/48	53.3	60/15
TCGA-50-6673-01	X	X	X	X	X	0.137	38.5	143/48	53.3	60/15
TCGA-55-6981-01	X	O	X	X	X	0.140	35.5	110/40	53.3	93/23
TCGA-38-4628-01	X	O	X	X	X	0.140	35.5	110/40	53.3	93/23
TCGA-55-6980-01	X	O	X	O	X	0.141	35.5	107/39	52.5	96/24

^a^“X”, denotes alteration to genome and/or transcriptome; “mo” month.

^b^Gene combination examined by Kaplan Meier Survival Plot for Entire LUAD cohort (Nature, 2014).

### Pathway alteration in EGFR activated LUAD

**Figure 7:**
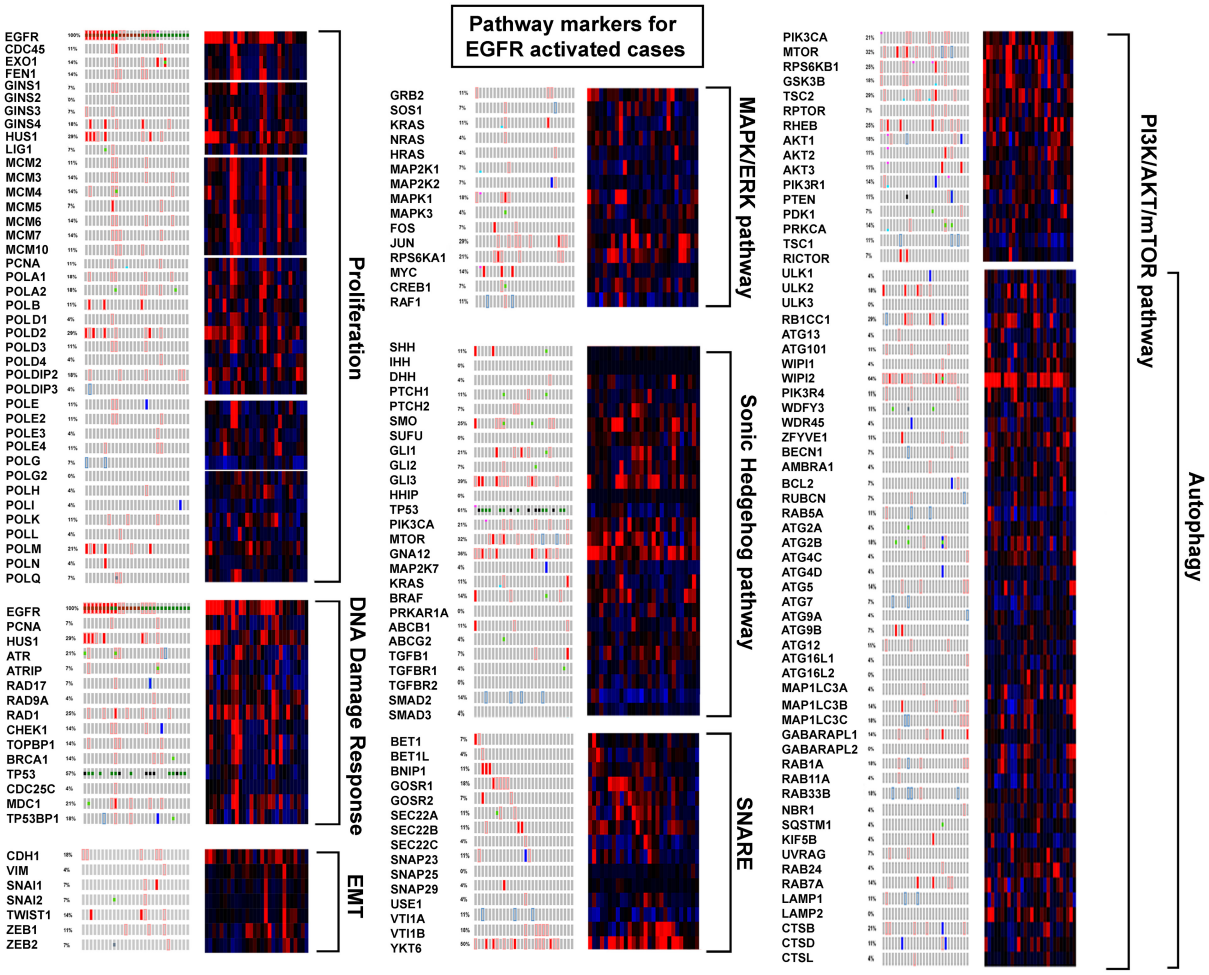
EGFR activated cases compared to pathway markers.

Overall, EGFR tyrosine kinase activated cases did not have distant metastases prompting examination of markers of epithelial to mesenchyme transition (EMT) (Figure 7). In agreement, appreciable alteration was not found for CDH1, VIM, SNAI1, SNAI2, TWIST1, ZEB1, or ZEB2. EGFR activation did correlate with activation of HUS1, RAD1, and several other components of the 9-1-1 DNA damage response pathway suggesting the major replication complexes were under replication stress. Important representative genes comprising the MAPK/ERK pathway revealed some activation of the pathway. The PI3K/AKT/mTOR pathway, particularly several components of autophagy, had high activation over a high frequency of cases. Examining the SHH pathway independently from other genes, found none of the components altered in 50% or over cases. However, GNA12 (7p22.3-p22.2) expression was upregulated and significantly co-expressed with EGFR (*P* = 0.001) in 36% of the EGFR activated cases, and Gli3 (7p14.1) expression in 39%.

## Discussion

This study shows expression of pre-replication and pre-initiation complex and replisome components vary between LUAD subtypes reported by TCGA [2, 3]. Subtypes 2 and 3 tumors have highest expression that potentiates highest proliferation. Subtype 1 tumors have the least of expression, and represent the opposite boundary. While cytotoxicity of cisplatin and carboplatin may be the result of complex cellular processes, it follows that DNA targeting agents would be most effective on highly proliferating tumors. And if so, the development of a proliferation descriptor based on component expression (or absence of expression for some genes) would be advantageous. An estimated measurement of the kinetics of proliferation could be investigated for correlation with responsiveness to DNA targeting reagents, possibly enabling prediction of response prior to use and permitting therapeutic decisions based on a score. Cases from LUAD subtype 1 and some patients with high EGFR expression and tyrosine kinase activating mutations for example, might benefit from less toxic therapies without prior cisplatin/carboplatin treatment. Both metastatic [10] and drug resistant tumor cells [11, 12] undergo a low/non proliferative phase that reactivates after extravasation in the case of metastasis, or clonal outgrowth in drug resistant cells. A standard form of measurement could be of help defining both processes.

EGFR is not a known component of the pre-replication and pre-initiation complexes or the replisome. It is a receptor tyrosine kinase (RTK) in the plasma membrane and functions in the regulation of the ERK/MAPK pathway kinase cascade and IGF-1 mTOR pathway [13]. Activating mutations are often found in the tyrosine kinase domain; RTK inhibitors are effective in LUAD treatment (for a recent review) [14]. That EGFR activation contributes to cancer is implicit from decades of research [15–18]. It is difficult to fathom that this gene’s over expression would not clearly correlate with increased replication processing. In actuality, despite the large body of work that exists, a full understanding of how or if the receptor functions in the proliferation process is lacking. EGFR is known to phosphorylate PCNA stabilizing the chromatin bound form [19], however the functional significance of the modification is not definitively clear. Some evidence exists that mismatch repair (MMR) is inhibited by EGFR phosphorylation of PCNA [20]. In the present study, the low proliferation status of highly expressed EGFR cases suggests PCNA phosphorylation by EGFR may actually play a role in slowing replication complexes. An alteration leading to over transcription of EGFR and surrounding chromosome 7 genes would be a germline or early event in these tumors, and PCNA phosphorylation would hypothetically result in “replication stress”, an early and strong driving force in tumorigenesis, which would then invoke the DNA damage response. Interestingly, transcription and replication start sites often coincide at nucleosome free regions with some transcription factors playing a role in proliferation [21]. The activation/overexpression of EGFR may initiate a transcriptional feedback inhibition mechanism for EGFR and replication genes. This study shows that tumors with missense activating EGFR mutations did not fall any one place on the PCNA proliferation curve, and (with one exception) EGFR activated cases were pre-metastatic. None-the-less, these untreated cases where EGFR activation was determined to be a putative driver had significant decreased survival on Kaplan Meier plots compared to cases that did not, suggesting that over proliferation and metastasis are not driving their lethality. In attempt to create a narrative of EGFR activated tumors, the remainder of this discussion goes into some detail explaining how the receptor was first associated with proliferation and then examines the functions of genes and pathways found altered in EGFR activated cases.

The earliest report pertinent to EGFR was written in 1947 on “urogastrone” known at the time as an inhibitor of gastric secretion, later identified as EGF [22, 23]. Initially, isolated from the submaxillary glands of male mice EGF elicited early eyelid opening and tooth eruption [24]. Embryonal chick epidermal sections incubated with purified EGF and radioactively labeled thymidine had increased nucleotide incorporation and keratinization, establishing EGF’s identity as proliferation factor. Curiously, control embryonal epidermal sections grown without EGF were capable of generating feathers while sections grown in the presence of purified EGF could not. Also, EGF stimulated basal cell production but the columnar orientation of the basal cells was not maintained. EGF did not stimulate the periderm, a structure present in chick embryonic skin made up of squamous cells linked closely together by junctional complexes and sloughed off at hatching [25]. Cohen [24] reported periderm contact with supporting filter led to migration of cells with no proliferation. In retrospect, the loss of columnar orientation observed in this early study is suggestive of an EGF/EGFR relationship to the cytoskeleton, abnormal keratinization hints at *Wnt* signaling involvement, and the disparity in feather formation and periderm behavior suggests that the EGF/EGFR relationship to proliferation is cell type dependent and complex. Molecular mechanisms of chick feather formation involve sonic hedgehog (Shh) [26] and the pathway is also thought involved in human lung development [27]; canonical activation is not thought to occur generally in LUAD. In this study EGFR activated LUAD had non-canonical Shh pathway activation through increased GNA12 and Gli3 expression (not Shh, Ptch, and Smo). Both of these genes are found on the short arm of Chromosome 7 where fifteen other transcriptionally dysregulated genes were identified suggesting the region is specifically important in EGFR activated lung cancer. Recent reports implicate the region in LUAD [28, 29].

This study found that with exception of TP53 and MCPH1, genes altered in EGFR cases were over expressed, NUDCD3 with highest frequency. NUDCD3 protein localizes to the cytoskeleton and is important in cytokinesis, mitosis, and dynein (cytoskeletal motor protein) stability. Over expression causes defects in cytokinesis that inhibit proliferation and induce the formation of binucleated cells, multipolar spindles, and lagging chromosomes [30]. Low TP53 expression and TP53 mutation was found in these tumors as was overexpression to FBXO42 [31] and UBE2D4 [32]. Both FBXO42 and UBE2D4 facilitate TP53 protein ubiquitination and degradation. TP53 absence would eliminate apoptosis, contribute to cell cycle arrest, alter angiogenesis, affect DNA repair, increase IGF-1/mTOR pathway signaling, and change exosome mediated secretion [33]. This study finds an increase in mTOR signaling in these cases, conjecturally a result of TP53 elimination and non-canonical hedgehog signaling (via GSK3β, also a member of *Wnt* signaling). PI3K/AKT/mTOR pathway favors tumor growth and cell size over proliferation and regulates autophagy. EGFR activated tumors have been suggested to be oncogene addicted to and dependent on this pathway [34, 35].

Autophagy, the endomembrane degradation process necessary for cellular homeostasis [36], has similarities and connections to apoptosis through BCL-2, an inhibitor of both. High EGFR expressing tumors had low BCL-2 expression in this study (data not shown) enabling increased autophagy in the absence of TP53 mediated apoptosis. Dysregulation of autophagy occurs in cancer, neurodegeneration, and microbial infection. This investigation observed autophagy components WIPI2 and YKT6 over expressed in EGFR activated tumors. WIPI proteins normally form a “propeller” that binds specifically to phosphatidylinositol 3-phosphate (PIP3, upregulated in mTOR/AKT/PI3K signaling) responsible for lipidation [37]. YKT6, a member of the SNARE proteins, is important in vesicular trafficking between the endoplasmic reticulum and the golgi apparatus and in neurotransmitter exocytosis [36]. Proteins involved in autophagy and exosome production, a process related through TP53, paradoxically contribute to tumor suppression in normal cells, and tumor promotion in cancer cells. Tumors with TP53 mutation and EGFR activation have altered exosome cargo currently being examined for biomarker use in liquid biopsies, effect on immune response, and cell-cell intercommunication. The dysregulation of these processes suggest possible involvement in the lethality of the EGFR activated phenotype.

Interestingly, WIPI2 like MCPH1 has a central nervous system phenotype (CNS) phenotype when mutated in humans [38, 39]. MCPH1 also functions in chromosome condensation [40], the DNA damage response, and regulation of CHK1 and BRCA1 [41] localizing to the centrosome in neurons [42]. Null MCPH1 in Drosophila undergo mitotic arrest with spindles that lack chromosomes, a phenotype that can be suppressed by Chk2 mutation [43]. MCPH1 is synonymous to BRIT1 [44], and is another post-transcriptional regulator of TP53 through ubiquitination. It is implicated in cancer as a tumor suppressor [45, 46, 47]. This study also suggests MCPH1 is a tumor suppressor in EGFR activated LUAD, however the lack of increasing significance in Kaplan Meier plots with additional affected genes (including TP53), and the decrease in median survival of the unaltered curves over total LUAD is perplexing (Table 8, Figure 6) possibly suggesting, like NUDCD3, a narrow window of expression is necessary to maintain mitotic capability or that DDR capability is necessary for EGFR activated tumor cells to replicate and survive.

When EGFR activated cases do metastasize, they target the brain at a much higher rate that non-EGFR activated cases. These tumor cells may bear some functional resemblance to the environment they are capable of metastasizing with both tumor and neuron being low proliferating with high EGFR expression and increased membrane trafficking. Some of the membrane trafficking components are known to play a role in neural synaptic transmission. These thoughts raise speculative questions, do unappreciated CNS symptoms exist in pre-metastatic EGFR activated LUAD patients? Could exosomes originating from EGFR activated tumors cross the blood brain barrier and interfere with neural synaptic transmission? Another question that may address EGFR activation lethality, do exosomes affect immune response in the lung?

## Materials and Methods

### Tumor cohort, cluster and mutational analysis

The tumor cohort used throughout this study was created and examined by TCGA [2, 3]; patient sample information is in Supplementary Table 3. Whole Exome Sequencing was performed on tumor and germline DNA. Cluster analysis based on copy number, DNA methylation, and mRNA expression revealed six subtypes (1-6) [4]. Mutational findings, also based upon data generated by the TCGA Research network, are found in cBio-Portal [6, 5, 48].

### Availability of data and materials

The datasets generated and/or analyzed for the current study are available in the Genome Data Commons [49] and Broad Institute [7].

### Expression analysis

“Differential expression” is defined as mRNA Z Scores (RNA-Seq by Expectation Maximization (RSEM) (log2)) compared to the expression distribution of each gene tumors that are diploid for the gene. The comparison employs the average expression level of the diploid tumor fraction as an estimated “normal” value, and was calculated by cBioPortal; values for all genes given (Supplementary Table 4).

In some instances, mRNA Z scores (RNA-Seq Reads per Kilobase of Transcript per Million (RPKM) (log2)) were also calculated, relative to the mean expression level of the tumor cohort in its entirety. This comparison facilitates bimodal placement of the gene’s expression level into high and low grouping around the tumor average for examination, it was described in more detail previously [50].

RNA-Seq derived exon expression levels were visualized in heat maps for all genes. The Gene Annotation File (GAF) “TCGA.hg19.June2011.gaf” [49] was used to create an exon-StartStop.txt file for each gene tested which in turn was used to parse the “UVM.rnaseqv2__illuminahiseq_rnaseqv2__unc_edu__Level_3__exon_quantification__data.da ta.txt” file [7] to create an “exonRPKM.txt” file used for standard Z score generation. Both files, exonStartStop.txt and exonsRPKM.txt, were run through a verification step to confirm that the appropriate gene, TCGA barcodes, and RNA-Seq data were selected prior to their use. Exon start-stop sites from the exonStartStop.txt file were examined in Integrative Genome Viewer (IGV) [51, 52] using RNA-Seq data from the same case to confirm the authenticity of the exon. Z scores were calculated for each exon of each gene by mean-centering with the average alteration level the log2 transformed RPKM values and dividing by the standard deviation, visualizing high (*red*), no change/no expression (*white*), and low (*blue*) and arranging data by LUAD cluster assignments (1–6) in heat maps.

The “Firebrowse” tool in Firehose [7] was used to examine differential expression levels of a specific gene across thirty-seven tumor types.

### Clinical data and survival analysis

The TCGA LUAD cohort was made up of two hundred-thirty matched tumor and normal samples from patients that did not have previous treatment. Appropriate informed consent was obtained. All major histologic types of lung adenocarcinoma were represented: 5% lepidic, 33% acinar, 9% papillary, 14% micropapillary, 25% solid, 4% invasive mucinous, 0.4% colloid and 8% unclassifiable adenocarcinoma. A full description of the pathological and histological assessment can be found in the Supplementary Materials of the TCGA report [2] (see also Supplementary Table 3). Kaplan Meier survival plots were constructed for “overall survival” and created either using the cBioPortal survival tool, or by using GraphPad Prism 6.0 software where indicated. In all cases the Log-rank (Mantel-Cox) and Hazard Ratio tests were used to determine significance. In some instances the Gehan-Breslow-Wilcoxon test which gives weight to deaths at early time points of the survival curve was also observed.

### Replication component expression as reference curve

To examine subtle relationships between gene expression and replication over the LUAD cohort, Proliferating Cell Nuclear Antigen (PCNA) and other components of the replisome were arranged from minimum and maximum expression and used as a reference curve. Test gene Z scores were arranged according to the reference gene case order using GraphPad Prism 6.0 software; data was obtained from cBioPortal unless otherwise indicated. The rationale for using PCNA in this manner stems from its integral role as a clamp in the replication process, to which many other proliferation factors bind [53]. It is used in this study as a replication marker.

### Identification of altered genes in EGFR activated cases

A list for “total genes” was generated [54] (approximately 22,165). Protein coding genes were screened for mutations, copy number alterations, mRNA expression (RNA Seq V2 RSEM), and protein expression (RPPA) in cBioPortal. Genes with alterations in at least 50% or more EGFR activated cases were identified. This gave extensive but not exhaustive results, RNA genes were not available for observation.

### Pathway analysis and reference sources

The Kyoto Encyclopedia of Genes and Genomes (KEGG) resource was used to examine the placement of specific genes in pathways [36]. Canonical pathways and networks were also examined using Metacore [55], references were examined in Metacore and PubMed.

## SUPPLEMENTARY MATERIALS











## Abbreviations

EGFR: Epidermal Growth Factor Receptor
TCGA: The Cancer Genome Atlas
LUAD: Lung Adenocarcinoma
CCLE: Cancer Cell Line Encyclopedia
COAD: Colon Adenocarcinoma
BRCA: Breast Cancer
GBB: Glioblastoma
MCM: Mini-Chromosome
EMT: Epithelial to Mesenchyme transition
SHH: Sonic Hedgehog
RTK: Receptor tyrosine kinase
MMR: Mismatch Repair
EGF: Epidermal Growth Factor
RSEM: RNA-Seq by Expectation Maximization
PCNA: Proliferating Cell Nuclear Antigen
KEGG: Kyoto Encyclopedia of Genes and Genomes
