# Supplementary material for: Modulation of proliferation factors in lung adenocarcinoma with an analysis of the transcriptional consequences of genomic EGFR activation
Source: Oncotarget. 2019 Dec 10;10(65):6913–33. doi: 10.18632/oncotarget.27316 (PMC6916753; doi:10.18632/oncotarget.27316)
Supplement: Supplementary file 1 [file oncotarget-10-6913-s001.pdf]

Modulation of proliferation factors in lung adenocarcinoma with an analysis of the transcriptional consequences of genomic EGFR activation

SUPPLEMENTARY MATERIALS

Supplementary Table 1: Survival data for EGFR activated cases. See Supplementary Table 1

Supplementary Table 2: List of cell lines from Cancer Cell Line Encyclopedia. See Supplementary Table 2

Supplementary Table 3: Clinical data from all LUAD cases. See Supplementary Table 3

Supplementary Table 4: Expression data for all genes examined. See Supplementary Table 4
